# Using formalin fixed paraffin embedded tissue to characterize the preterm gut microbiota in necrotising enterocolitis and spontaneous isolated perforation using marginal and diseased tissue

**DOI:** 10.1186/s12866-019-1426-6

**Published:** 2019-03-04

**Authors:** Christopher J. Stewart, Roxana Fatemizadeh, Pamela Parsons, Christopher A. Lamb, Deborah A. Shady, Joseph F. Petrosino, Amy B. Hair

**Affiliations:** 1 Alkek Center for Metagenomics and Microbiome Research, Department of Molecular Virology and Microbiology, Baylor College of Medicine, Houston, TX USA; 2 Institute of Cellular Medicine, Newcastle University, Medical School, Leech Building, M3.121, Newcastle upon Tyne, NE2 4HH UK; 3 Section of Neonatology, Department of Pediatrics, Baylor College of Medicine, Texas Children’s Hospital, Houston, TX USA; 4 Texas Medical Center Digestive Diseases Center, Core B Cellular and Molecular Morphology, Houston, TX USA; 5 Newcastle upon Tyne Hospitals NHS Foundation Trust, Newcastle upon Tyne, UK; 6 Department of Pathology and Immunology, Baylor College of Medicine, Houston, TX USA

**Keywords:** Preterm, Gut microbiome, Necrotising enterocolitis, Spontaneous intestinal perforation, Formalin-fixed paraffin-embedded, Tissue

## Abstract

**Background:**

Necrotising enterocolitis (NEC) is a common cause of death in preterm infants and is closely linked to the gut microbiota. Spontaneous intestinal perforation (SIP) also occurs in preterm neonates, but results in lower mortality and less adverse neonatal outcomes than NEC. Existing studies are largely limited to non-invasive stool samples, which may not be reflective of the anatomical site of disease. Therefore, we analysed historical formalin-fixed paraffin-embedded (FFPE) tissue from NEC and SIP preterm infants. A total of 13 NEC and 16 SIP infants were included. Extracted DNA from FFPE tissue blocks underwent 16S rRNA gene sequencing. For a subset of infants, diseased tissue and marginal healthy tissue from the same infant were compared.

**Results:**

Xylene provided a cost and time effective means of deparaffinization. Tissue from the site of disease was highly comparable to adjacent healthier tissue. Comparing only diseased tissue from all infants showed significantly lower Shannon diversity in NEC (*P* = 0.026). The overall bacterial communities were also significantly different in NEC samples compared to SIP (*P* = 0.038), and large variability within NEC infants was observed. While no single OTU or genus was significantly associated with NEC or SIP, at the phylum level Proteobacteria (*P* = 0.045) and Bacteroidetes (*P* = 0.024) were significantly higher in NEC and SIP infants, respectively.

**Conclusions:**

Existing banks of intestinal FFPE blocks provide a robust and specific sample for profiling the microbiota at the site of disease. We showed preterm infants with NEC have lower diversity and different bacterial communities when compared to SIP controls.

**Electronic supplementary material:**

The online version of this article (10.1186/s12866-019-1426-6) contains supplementary material, which is available to authorized users.

## Background

Necrotising enterocolitis (NEC) and spontaneous intestinal perforation (SIP) represent the two major types of intestinal perforation in preterm neonates. While both conditions are associated with prematurity, NEC and SIP are separate clinical and pathological entities [1]. NEC typically involves coagulative necrosis and spans a larger area of intestine, whereas SIP is associated with hemorrhagic necrosis involving a small focal lesion with adjacent histologically normal mucosa [2]. Because development of NEC results in increased mortality and higher risk of adverse neonatal outcomes when compared to SIP [1, 3, 4], NEC can be considered a more serious intestinal disorder for preterm infants.

Abnormal bacterial colonisation and an immature immune response are among the main risk factors for NEC. Clinical investigations exploring associations between preterm gut bacterial communities (termed the microbiota) and NEC have yielded inconsistent findings. Overall, taxonomic comparisons at the phylum level tend to show the most concordance between different studies, with Proteobacteria higher in NEC infants prior to disease diagnosis [5–10]. Taxonomic comparisons at the genus level are less conclusive, with no single causative genus reported consistently within and between studies, although *Bifidobacterium* may play important roles in protection from disease [6, 11–13]. Taxonomic comparisons beyond genus are less explored, but the advent of metagenomic sequencing is beginning to elucidate associations at the species and strain level. For instance, higher relative abundance of uropathogenic *Escherichia coli* and a lower relative abundance of species associated with older infants (e.g., *Veillonella* spp.) were found prior to NEC diagnosis [14]. There are no reported associations of other taxonomic kingdoms with NEC, such as fungi, which have low viability in the preterm gut [15, 16]. Existing literature regarding diversity is also inconclusive, with some reports describing a reduced diversity in NEC [7, 10, 17–20], while others found no significant difference between infants with NEC and controls [6, 21–23].

The vast majority of investigations of the preterm gut microbiome have used stool samples as a non-invasive proxy for bacterial colonisation of the small intestine. However, three existing studies have analyzed disease tissue directly. A study of frozen tissue resections from 16 NEC and 10 non-NEC samples supported results from stool based studies, showing NEC tissue generally had higher bacterial load but lacked a specific causative agent [10]. A separate study analysing only NEC samples from formalin-fixed paraffin-embedded (FFPE) tissue detected bacteria in 22/24 samples, with dominance by the Proteobacteria phyla [24]. A third study using FFPE ileum tissue reported a lack of pathogens in the preterm gut based on a specific multiplex PCR amplification array [25].

FFPE tissue offers a novel means of exploring the preterm gut microbiota in NEC and non-NEC conditions, advancing on existing stool studies to ascertain the community at the site of disease. In the current study, we used historical FFPE samples from NEC and SIP (to serve as non-NEC controls) preterm infants. We aimed to 1) establish a suitable DNA extraction procedure for FFPE blocks, 2) compare tissue from the site of disease and adjacent healthier tissue, and 3) determine associations between the microbiota of preterm infants diagnosed with NEC compared to SIP controls.

## Results

### Microbiota profiles were comparable regardless of the solution used for deparaffinization

Comparison of xylene and ‘Deparaffinization Solution’ showed high comparability in the resulting microbiota profiles within patients (Additional file 1: Figure S1). The Shannon diversity showed no difference (*P* = 0.80; Additional file 1: Figure S1A) and weighted UniFrac PCoA demonstrated each sample was comparable, regardless of the deparaffinization method (Additional file 1: Figure S1B). Heatmap analysis further showed that samples grouped independent of deparaffinization method and that the identified OTUs were comparable within patients (Additional file 1: Figure 1SC). Thus, xylene was used for all extractions owing to reduced cost and faster extraction time of this solution.

**Fig. 1:**
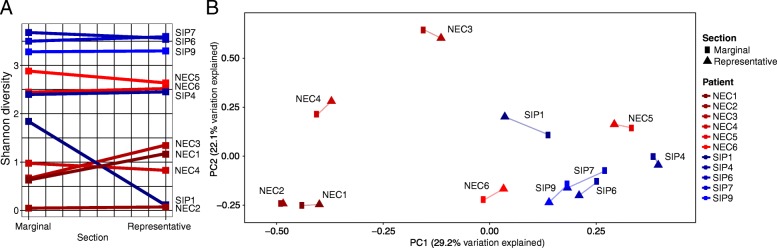
Comparison of representative tissue collected at the site of disease and marginal tissue collected from adjacent healthier tissue. A subset of 11 infants with multiple tissue sections were included in the analysis. Of the 11 infants, six were diagnosed with necrotising enterocolitis (NEC) and five were diagnosed with spontaneous intestinal perforation (SIP). Matched samples are connected in each plot. **a**) Shannon diversity between matched representative and marginal sections. **b**) Weighted UniFrac principal coordinate analysis (PCoA)

### Characterisation of intestinal microbiota obtained from FFPE samples

The microbiota from preterm FFPE intestinal samples collected from NEC or SIP infants showed four distinct taxonomic profiles; 1) *Escherichia* dominant, 2) *Klebsiella* dominant, 3) *Staphylococcus* dominant, and 4) mixed profile with relatively high *Bacteroides* (Additional file 2: Figure S2). The Shannon diversity was highly comparable between representative and marginal sections (*P* = 0.92), although in one infant (SIP1) the diversity showed a large reduction between the marginal and representative sample (Fig. 1a). Additionally, weighted UniFrac PCoA showed high concordance of the bacterial community within patients, regardless of whether the tissue was obtained at the site of disease or an adjacent healthier section (Fig. 1b).

MaAsLin analysis was performed on all available metadata categories (Table 1) to determine the significant associations between OTUs and metadata. Only the location of insult had a significant association with any OTU, with *Lachnoanaerobaculum* higher in colonic samples and *Neisseria* lower in ileum samples (*P* = < 0.001; Additional file 3: Table S1). These associations should be interpreted with consideration that the location of insult metadata category had only 2 colonic samples and 2 jejunum samples, and 25 ileum samples, and because the significant OTUs comprised low overall relative abundance (Additional file 4: Table S2). No OTU was significantly associated with NEC or SIP.

**Table 1:** Patient characteristics of infants with necrotising enterocolitis or spontaneous intestinal perforation

	NEC	SIP	*P* value
Number of samples	13	16	–
Gestation (weeks), median (IQR)	25 (24–28)	25 (24–27)	0.888
Birth weight (g), median (IQR)	731 (610–794)	718 (688–790)	0.257
Vaginal birth	4 (31%)	4 (25%)	0.999
Male	8 (62%)	12 (75%)	0.707
Diagnosis (DOL), median (IQR)	20 (18–35)	6 (5–8)	0.066
Surgery (DOL), median (IQR)	30 (22–42)	7 (6–10)	0.079
Section			0.975
Jejunum	1 (8%)	1 (6%)	
Ileum	11 (85%)	14 (88%)	
Colon	1 (8%)	1 (6%)	
Bacteraemia prior to surgery	7 (54%)	0 (0%)	0.003
Any antibiotics prior to surgery	13 (100%)	15 (94%)	0.999
Days antibiotics prior to surgery, median (IQR)	8 (3–17)	2 (1–3)	0.257

Data are no. (%) of infants unless otherwise indicated

Abbreviation: *DOL* day of life, *IQR* interquartile range, *NEC* necrotising enterocolitis, *SIP* spontaneous intestinal perforation

### Microbiota profiles of intestinal tissue is significantly different between NEC and SIP infants

**Fig. 2:**
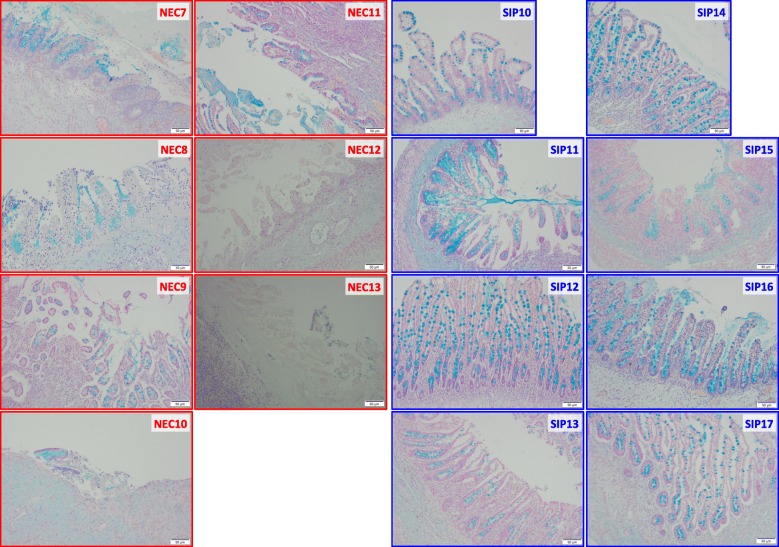
Histology of representative sections from infants with necrotising enterocolitis (NEC) and spontaneous intestinal perforation (SIP). Sections stained with Alcian blue to visualize the goblet cells (shown in blue), as a measure of epithelial integrity. The segment from each sample with the most normal villous architecture is shown

**Fig. 3:**
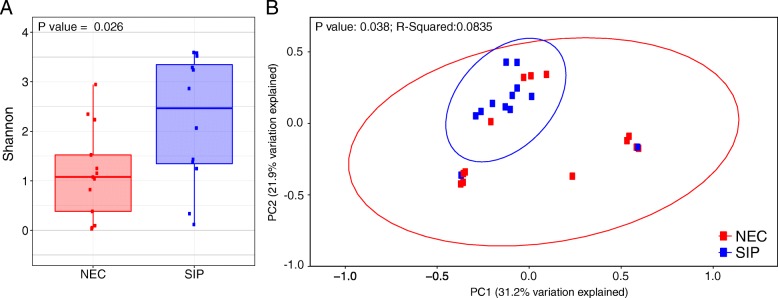
Alpha- and beta-diversity analysis of preterm infants diagnosed with necrotising enterocolitis (NEC) and spontaneous intestinal perforation (SIP). Only representative sections were included in the analysis. **a**) Shannon diversity between NEC and SIP representative sections. **b**) Bray-Curtis principal coordinate analysis (PCoA). Ellipses represent the 95% confidence intervals

Histological analysis of representative tissue sections demonstrated the widespread architectural distortion with variable mucosal ischemic necrosis, inflammatory cell infiltrate and epithelial destruction with loss of goblet cells associated with NEC, in comparison to the relatively preserved villous architecture, crypt morphology and epithelium seen in SIP without mucosal ischemic necrosis (Fig. 2). Microbiota analysis of only representative sections from all infants (13 NEC vs. 16 SIP infants) showed NEC infants had significantly lower Shannon diversity compared to SIP infants (*P* = 0.026) (Fig. 3a). The mean Shannon diversity of NEC infants was ~ 1.0, whereas the mean Shannon diversity of SIP infants was ~ 2.5. Additionally, the microbiota profiles were significantly different between NEC and SIP infants based on weighted UniFrac PCoA (*P* = 0.038) (Fig. 3b). In comparison to SIP infants, the bacterial communities in NEC infants were highly dissimilar and showed large variance between infants, as demonstrated by the much larger 95% confidence interval eclipses in the NEC group. Only two SIP infants clustered outside of the 95% confidence interval for this group and these two samples showed the lowest Shannon diversity of all SIP samples. No metadata category correlated to the low diversity in these two samples. Specifically, one infant was female, vaginally delivered, 2270 g birth weight, and had surgery on day 5, whereas the other infant was male, caesarian delivered, 689 g birth weight, and had surgery on day 18.

**Fig. 4:**
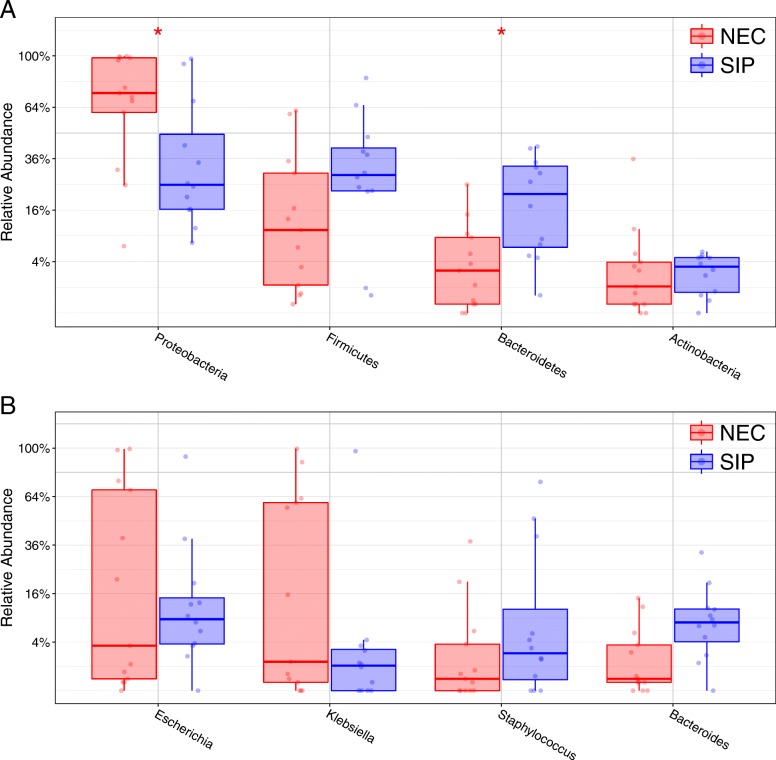
Box plots showing the relative abundance of the most dominant bacterial phyla and genera between preterm infants diagnosed with necrotising enterocolitis (NEC) and spontaneous intestinal perforation (SIP). Taxa > 0.05% minimum relative average abundance included. **a**) Phyla level analysis. Proteobacteria was significantly higher in NEC infants (*P* = 0.045) and Bacteroidetes was significantly higher in SIP infants (*P* = 0.024). **b**) Genera level analysis. No genus was significantly associated with NEC or SIP

Statistical analysis of bacterial phylum showed Proteobacteria relative abundance was significantly higher in NEC infants (*P* = 0.045), whereas Bacteroidetes relative abundance was significantly higher in SIP infants (*P* = 0.024) (Fig. 4a and Additional file 5: Figure S3). Statistical analysis of bacterial genera showed no significant difference between NEC and SIP infants (Fig. 4B), supporting the previous OTU level MaAsLin modelling.

## Discussion

The vast majority of investigations of the preterm gut microbiome have used stool samples, which are typically representative of the distal colon, but NEC most typically occurs in the ileum [26]. In the current study, we analyzed the bacterial community in FFPE intestinal tissue resected from preterm infants diagnosed with NEC or SIP. NEC is a serious intestinal disorder [1, 3, 4], whereas SIP is a focal defect in an otherwise normal intestine. Thus, SIP infants provided tissue as close as feasibly possible to ‘healthy’ control tissue in neonates and served as a suitable control group for NEC patients given their demographic similarity. We confirmed the suitability of SIP to serve as a control comparator histologically, with preserved small bowel architecture without the necrosis and tissue destruction noted in NEC. We found high comparability in the microbiota within patients when evaluating tissue from the site of disease relative to adjacent healthier tissue. Comparing only diseased tissue from NEC and SIP infants showed lower Shannon diversity and greater microbiota dissimilarity between NEC infants. While no single OTU or genus was significantly associated with NEC or SIP, at the phylum level Proteobacteria and Bacteroidetes were significantly higher in NEC and SIP infants, respectively.

Only three existing studies have explored the microbiota in preterm resected tissue, one using frozen tissue [10] and two using FFPE samples [24, 25]. In both FFPE studies, only NEC samples were included (i.e., no non-NEC controls), precluding any statistical comparison between NEC and non-NEC disease. Additionally, both FFPE studies used targeted sequencing of specific microbes, whereas the current study employed next generation sequencing using universal primers to more comprehensively profile the microbiota. Nonetheless, in agreement with Smith et al. (2011) we found high relative abundance of the Proteobacteria phylum in NEC infants [24], which was significantly higher when compared to SIP infants. Indeed, the phylum level analysis of tissue sections was in accordance with the results from a large meta-analysis of stool from NEC and non-diseased controls, with increased relative abundances of Proteobacteria and decreased relative abundances of Firmicutes and Bacteroidetes prior to NEC onset [5]. Furthermore, we found no single OTU or genus to be consistently associated with infants diagnosed with NEC, which is in accordance with all three tissue based studies [10, 24, 25] and other works in stool [5, 6, 18, 27, 28]. In the absence of specific causative organisms in NEC, it is fascinating that the Proteobacteria is consistently higher in NEC infants prior to disease diagnosis [5–10]. However, it is not possible to discern cause or effect based on the current data. For instance, Proteobacteria comprises mainly facultative organisms and the higher relative abundance of this phylum in NEC may reflect inflammation and oxidative-stress related factors underlying the disease (i.e., Proteobacteria better tolerate the potentially higher oxygen levels in NEC) [29]. Additionally, *Klebsiella* and *Escherichia* together make up the vast majority of the Proteobacteria phylum in preterm infants [5, 29], and these genera have been linked to the neonatal intensive care unit (NICU) environment [30]. While NICU-associated bacteria undergo rapid decline following discharge from hospital [31], correcting the abnormal bacterial colonisation during the initial weeks of life with pre- and pro-biotics remains an exciting and tangible opportunity.

Beyond specific bacterial taxa, there is evidence that the overall gut microbiota profiles and community dynamics are different between NEC and controls. This concept was not explored in any of the existing NEC tissue studies, but in the current study we observed significantly different microbiota profiles between NEC and SIP infants. Additionally, the overall microbiota profiles of SIP infants were highly comparable, whereas samples from NEC infants showed much greater variance, suggesting a less uniform pattern of microbial colonisation. This has been demonstrated in previous work analysing extensive longitudinal stool data, showing infants with NEC exhibited more dynamic microbiota development prior to disease onset, when compared to matched non-diseased controls [6]. It is intriguing that NEC infants in the current analysis, as well as previous tissue [10] and stool work [5, 6, 18], generally show reduced diversity prior to disease onset. Thus, this reduction in diversity may account for the reduced stability observed in NEC infants during disease progression. This is supported by existing knowledge that gut microbiota diversity increases throughout infancy, correlating with increased microbiota stability and resilience [32–34].

A novel aspect of the current study was the analysis of a disease (representative) and healthier (marginal) sections of the intestine from within the same individual. This showed the bacterial diversity and overall microbiota profiles were generally consistent within each infant between sections of tissue. Given the close proximity of the different tissue sections for each infant, this result is not surprising. Nonetheless, this finding informs future work in preterm infants that in the event the representative disease section is not available, investigators may use adjacent marginal tissue to accurately characterise the microbiota.

While the dominant taxa in the current study of tissue are comparable to the dominant taxa in stool from preterm infants, additional work is needed to determine how representative stool samples are of the site of disease using matched stool and tissue from the same infant. Further work should also compare fresh tissue to FFPE sections to ascertain differences and potential biases introduced during the process of fixing and embedding tissue. Notably, all samples were collected within an eight-year time frame (October 2009 – September 2017) using the same fixation protocol and storage conditions.

The current study has some limitations. First, the sample size of 13 NEC and 16 SIP infants is likely underpowered to detect slighter changes between groups. However, differences could be detected in a cohort of 13 NEC infants and the current cohort largely comparable in size to published works. Second, because we are working with tissue and not non-invasive samples (e.g., stool) we were unable to perform longitudinal analysis (no babies in the current cohort had multiple surgeries with resected tissue). We were also unable to include a ‘healthy’ non-disease control population (because they would not have surgery). Thus, in order to provide a comparator group for NEC we included infants who underwent surgery for SIP. Other non-NEC disorders could result in intestinal surgery (e.g., intestinal atresia, malrotation with volvulus, and imperforate anus), but we selected only SIP infants to serve as non-NEC controls in order to circumvent potential variability in the control group resulting from differing pathologies. As would be expected, the SIP group was generally younger (*P* = 0.079) and some of the NEC infants were also diagnosed with bacteremia (*P* = 0.003), which may influence the microbiota profiles. While we removed the only infant not to receive antibiotics prior to surgery, we did not analyze the effects of different antibiotic regimes on the microbiota. Given the high variability between each patients’ antibiotic regimes and the inability to collect longitudinal tissue samples, such work is outside the scope of the current analysis. Lastly, this observational study sought only to determine associations between tissue microbiota and preterm disease, thus no mechanism for disease is presented. Future work should employ models for investigating the role of the microbiota-host cross talk in NEC. For instance, the capacity to co-culture bacteria with primary cell lines (enteroids) from preterm infants holds huge promise to understanding disease mechanism and testing potential therapeutics [35].

## Conclusions

We successfully generated reproducible microbiota profiles from FFPE samples of preterm infants with NEC and SIP. We found that NEC infants had significantly different bacterial communities, with lower diversity and more diverse inter-individual microbiota profiles. No OTU or genera was associated with either NEC or SIP, but Proteobacteria and Bacteroidetes phyla were significantly higher in NEC and SIP infants, respectively. Because FFPE samples are generated during routine pathology from preterm gut resections and can be obtained retrospectively, there exists huge potential for investigators to study gut microbiota in already banked samples that allow for profiling at the site of disease.

## Methods

### Study design and cohort

The cohort consisted of 29 infants who required surgery at Texas Children’s Hospital from 2009 to 2017 13 underwent surgery for confirmed NEC and 16 underwent surgery for SIP. Representative FFPE sections (i.e., at the site of disease) were obtained for all 29 patients. Marginal sections (i.e., non-diseased tissue adjacent to the site of disease) were obtained from a subset of 11 patients of which six were NEC infants (NEC 1–6) and five were SIP infants (SIP 1, 4, 6, 7, 9). A separate subset of samples was used for histology from seven NEC infants (NEC 7–13) and eight SIP infants (SIP 10–17).

Overall the clinical variables between NEC and SIP infants were comparable, with no significant difference in the gestational age, birth weight, sex, diagnosis day of life (DOL), and surgery DOL (Table 1). All infants except one SIP infant received antibiotics prior to surgery, with no difference in antibiotic duration between NEC and SIP. The one SIP infant with no antibiotics was removed from the NEC vs. SIP analysis. Most tissue samples were obtained from ileum sections (85% in NEC and 88% in SIP). Bacteremia prior to onset was only diagnosed in NEC infants (54% in NEC and 0% in SIP; *P* = 0.003). Full per patient demographic information is available in Additional file 6: Table S3.

### DNA extraction

For each sample, the first few scrolls from the FFPE blocks were discarded and then eight 10 μm scrolls were cut and placed into sterile 2 ml centrifuge tubes. To test the most effective solution for deparaffinization of FFPE samples, we compared xylene (Sigma-Aldrich, MO, USA) and ‘Deparaffinization Solution’ (QIAGEN, CA, USA). After initial experimentation on 7 samples (NEC 1–4 and SIP 1–3), where scrolls from the same sample underwent paraffin removal with each solution, we used xylene for all remaining extractions (see results). DNA was extracted using the QIAamp DNA FFPE tissue kit (QIAGEN, CA, USA) per the manufacture’s protocol. Nucleic acids were eluted in 50 μl elution buffer following a 5 min on column incubation at room temperature. The microtome was cleaned with DNA AWAY (Thermo Scientific, MA, USA) between each sample and the equipment was regularly tested using sterile swabs that also underwent sequencing as controls.

### 16S rRNA gene sequencing

The bacterial 16S rRNA gene V4 region was amplified by PCR using barcoded Illumina adapter-containing primers 515F and 806R [36] and sequenced with the 2 × 250 bp cartridges in the MiSeq platform (Illumina, CA, USA). The read pairs were demultiplexed and reads were merged using USEARCH v7.0.1090 [37]. Merging allowed zero mismatches and a minimum overlap of 50 bases, and merged reads were trimmed at the first base with a Q ≤ 5. A quality filter was applied to the resulting merged reads and those containing above 0.5% expected errors were discarded. Sequences were stepwise clustered into Operational Taxonomic Units (OTUs) at a similarity cutoff value of 97% using the UPARSE algorithm [38]. Chimeras were removed using USEARCH v7.0.1090 and UCHIME. To determine taxonomies, OTUs were mapped to a version of the SILVA Database [39] containing only the 16S V4 region using USEARCH v7.0.1090. Abundances were recovered by mapping the merged reads to the UPARSE OTUs. A rarefied OTU table was constructed from the output files generated in the previous two steps for downstream analyses of alpha-diversity, beta-diversity (including UniFrac), and phylogenetic trends [40].

### Histology

Specimens for histological examination were fixed in 10% neutral buffered formalin, and processed for paraffin embedding. Sections were cut at 3 μm and stained with Alcian Blue pH 2.5 (Poly Scientific R&D Corp). An Olympus BX43 light microscope was used for imaging of histology sections and all images were taken at 10x magnification. Representative images were captured from every sample to demonstrate the widespread necrosis of NEC and the relatively normal small bowel histology seen in SIP.

### Statistical analysis

Samples were rarefied to 870 reads and rarefaction resulted in the loss of all five control swabs. After the initial analysis of eight samples deparaffinized with either xylene or ‘Deparaffinization Solution’, all samples extracted using ‘Deparaffinization Solution’ were removed from subsequent analysis. After the analysis of representative and marginal sections from the same infant, subsequent analysis comparing NEC and SIP included only the representative sections (and the marginal sections were omitted), in order to avoid repeated measures.

MaAsLin was used for adjustment of covariates when determining the significance of OTUs contributing to a specific variable, while accounting for potentially confounding covariates [41]. Briefly, this multivariate linear modelling system for microbial data selects from among a set of (potentially high-dimensional) covariates to associate with microbial taxon or pathway abundances. Mixed effects linear models using a variance-stabilizing arcsin square root transform on relative abundances are then used to determine the significance of putative associations from among this reduced set. Nominal *p*-values across all associations are then adjusted using the Benjamini-Hochberg false discovery rate (FDR) method. MaAsLin modelling was performed on all covariates presented in Table 1, with the exception of “Any antibiotics prior to surgery” owing to only a single infant being negative for this covariate. The default MaAsLin parameters were applied (maximum percentage of samples NA in metadata 10%, minimum percentage relative abundance 0.01%, *P* value < 0.05, q value < 0.25). All *P* values were adjusted for multiple comparisons using FDR [42].

Analysis and visualization of bacterial communities was conducted in R [43]. Significance of categorical variables were determined using the non-parametric Mann-Whitney test [44]. Principal coordinate analysis (PCoA) ordination plots were generated based on weighted (i.e., relative abundance) and unweighted (i.e., presence/absence) UniFrac [40]. Statistically significant differences between groups in PCoA were determined by non-parametric multivariate permutational multivariate analysis of variance (PERMANOVA) [45]. All *P*-values were adjusted for multiple comparisons with the FDR algorithm [42].

## Additional files

Additional file 1: **Figure S1.** Comparison of resulting microbiota profiles following DNA extraction with either xylene or ‘Deparaffinization Solution’ for removal of paraffin. A) Shannon diversity. B) Weighted UniFrac principal coordinate analysis (PCoA). C) Heatmap analysis showing the 10 most abundant genera. Heatmap intensity based on the relative abundance in each sample, normalised per row. (PDF 224 kb)

Additional file 2: **Figure S2.** Stacked bar plot of dominant genera. Marginal and representative sections from each infant were included separated by necrotising enterocolitis (NEC) and spontaneous intestinal perforation (SIP). (PDF 176 kb)

Additional file 3: **Table S1.** MaAsLin analysis of all available metadata categories to determine the significant associations between OTUs and metadata (XLSX 37 kb)

Additional file 4: **Table S2.** Relative abundance of significant genera as determined by MaAsLin analysis, per section of tissue. (XLSX 33 kb)

Additional file 5: **Figure S3.** Stacked bar plot of dominant phyla. Marginal and representative sections from each infant were included separated by necrotising enterocolitis (NEC) and spontaneous intestinal perforation (SIP). (PDF 169 kb)

Additional file 6: **Table S3.** Complete demographic information per patient. (XLSX 47 kb)

## Abbreviations

FDR: False discovery rate
FFPE: Formalin fixed paraffin embedded
NEC: necrotising enterocolitis
NICU: Neonatal intensive care unit
OTU: Operational taxonomic unit
PCoA: Principal coordinate analysis
PERMANOVA: Non-parametric multivariate permutational multivariate analysis of variance
SIP: Spontaneous intestinal perforation
