# Supplementary figures and images for: Using formalin fixed paraffin embedded tissue to characterize the preterm gut microbiota in necrotising enterocolitis and spontaneous isolated perforation using marginal and diseased tissue
Source: BMC Microbiol. 2019 Mar 4;19:52. doi: 10.1186/s12866-019-1426-6 (PMC6398254; doi:10.1186/s12866-019-1426-6)

A

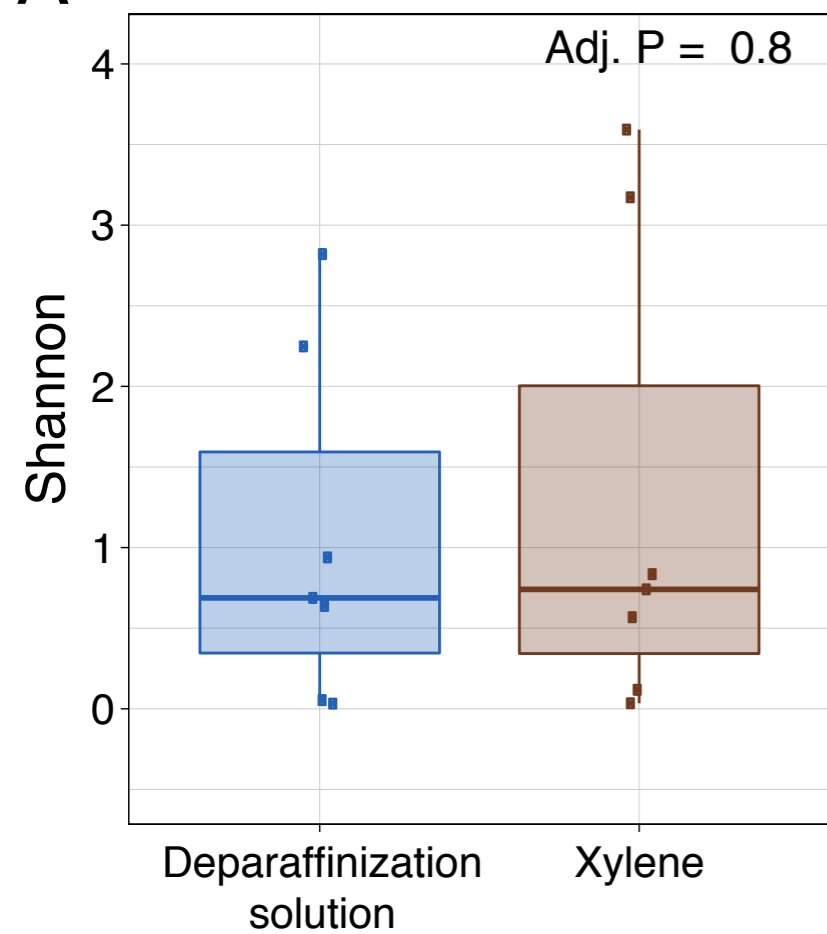

B

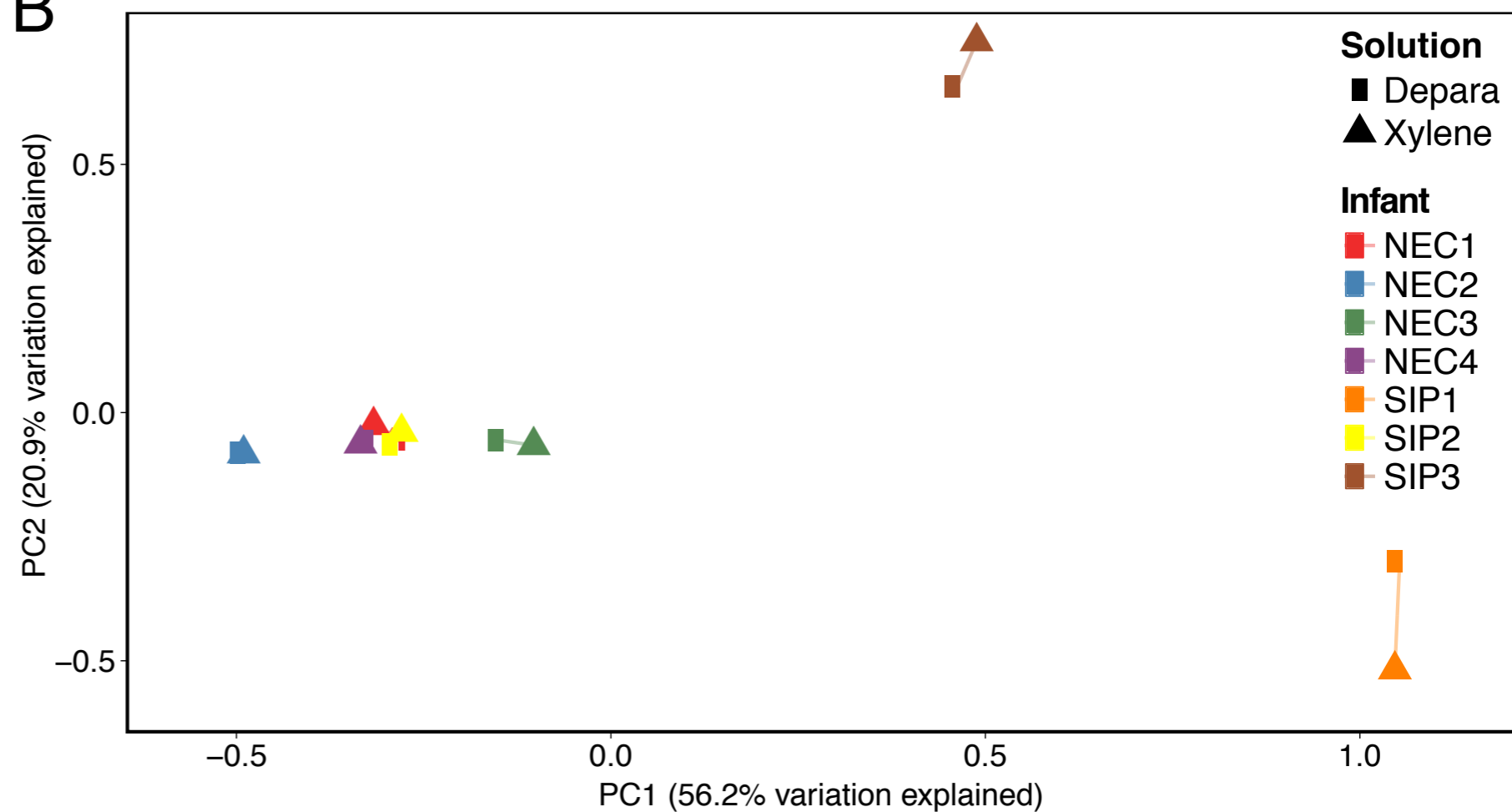

C

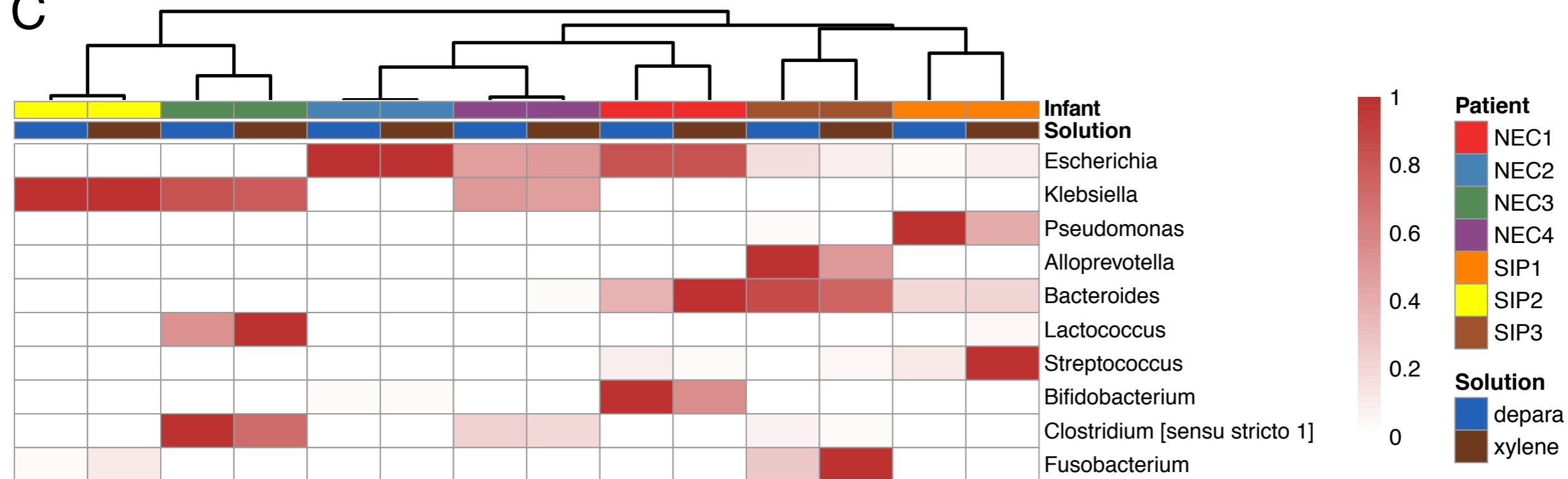

Supplement: Supplementary file 1 — Figure S1. Comparison of resulting microbiota profiles following DNA extraction with either xylene or ‘Deparaffinization Solution’ for removal of paraffin. A) Shannon diversity. B) Weighted UniFrac principal coordinate analysis (PCoA). C) Heatmap analysis showing the 10 most abundant genera. Heatmap intensity based on the relative abundance in each sample, normalised per row. (PDF 224 kb) [file 12866_2019_1426_MOESM1_ESM.pdf]

Relative Abundance

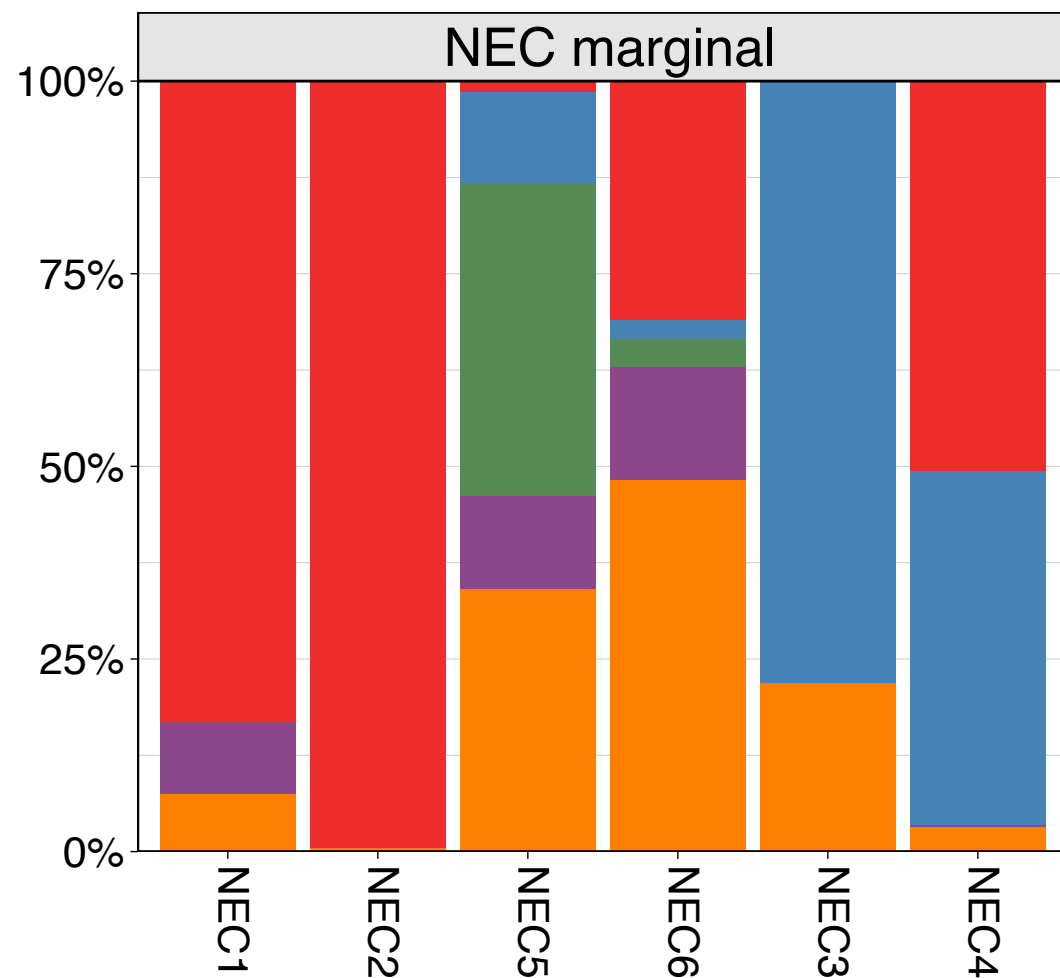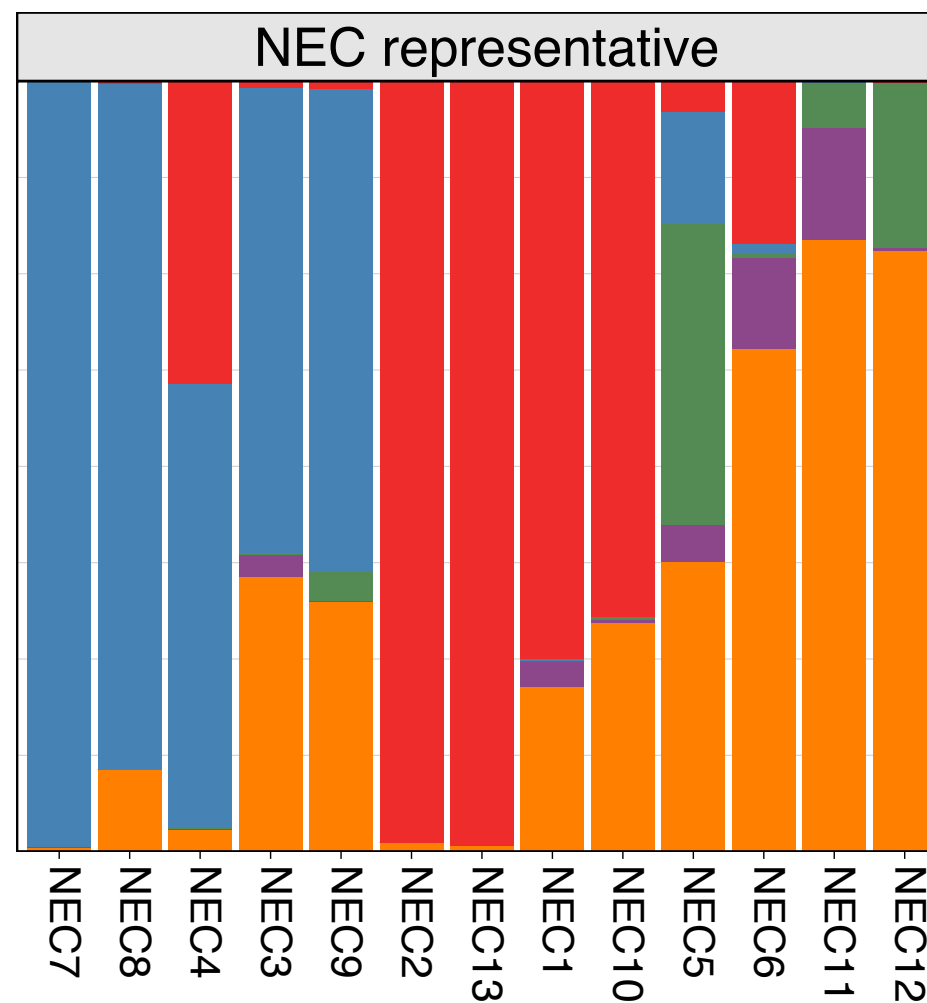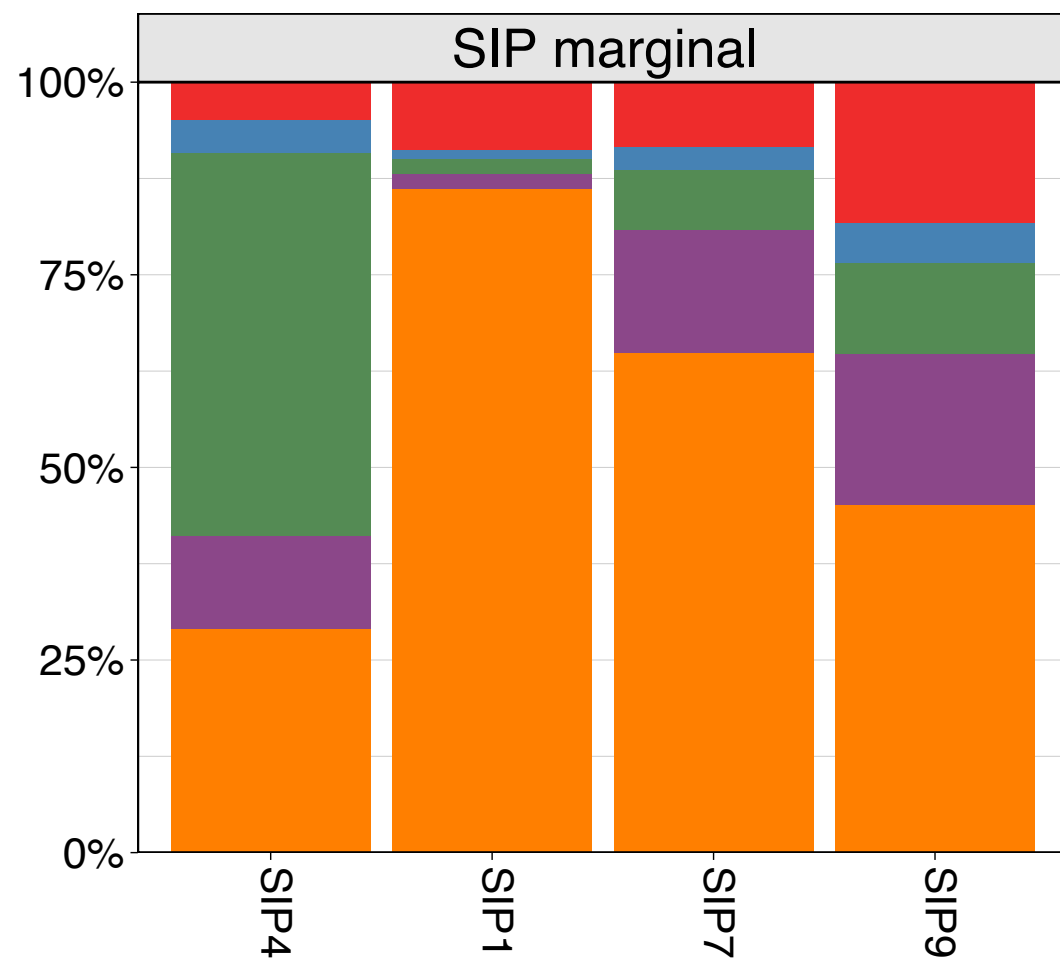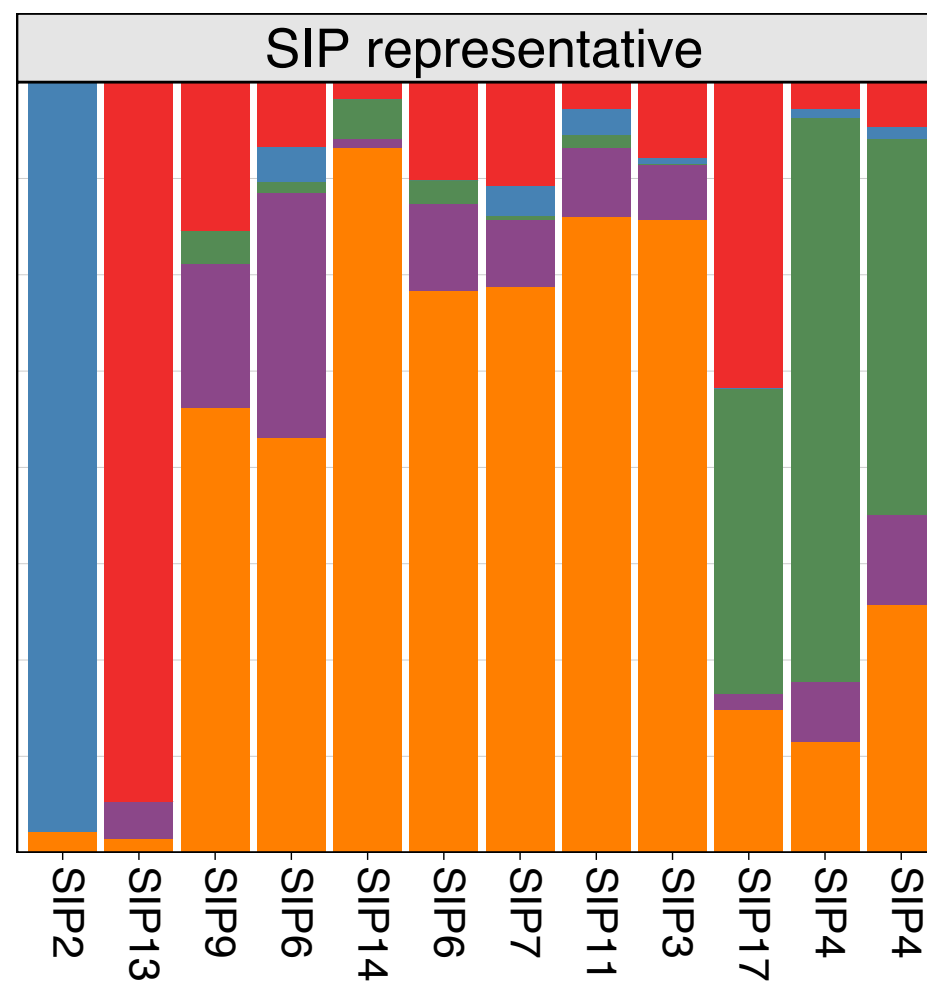

**Genus**

- Escherichia
- Klebsiella
- Staphylococcus
- Bacteroides
- Other

Supplement: Supplementary file 2 — Figure S2. Stacked bar plot of dominant genera. Marginal and representative sections from each infant were included separated by necrotising enterocolitis (NEC) and spontaneous intestinal perforation (SIP). (PDF 176 kb) [file 12866_2019_1426_MOESM2_ESM.pdf]

Relative Abundance

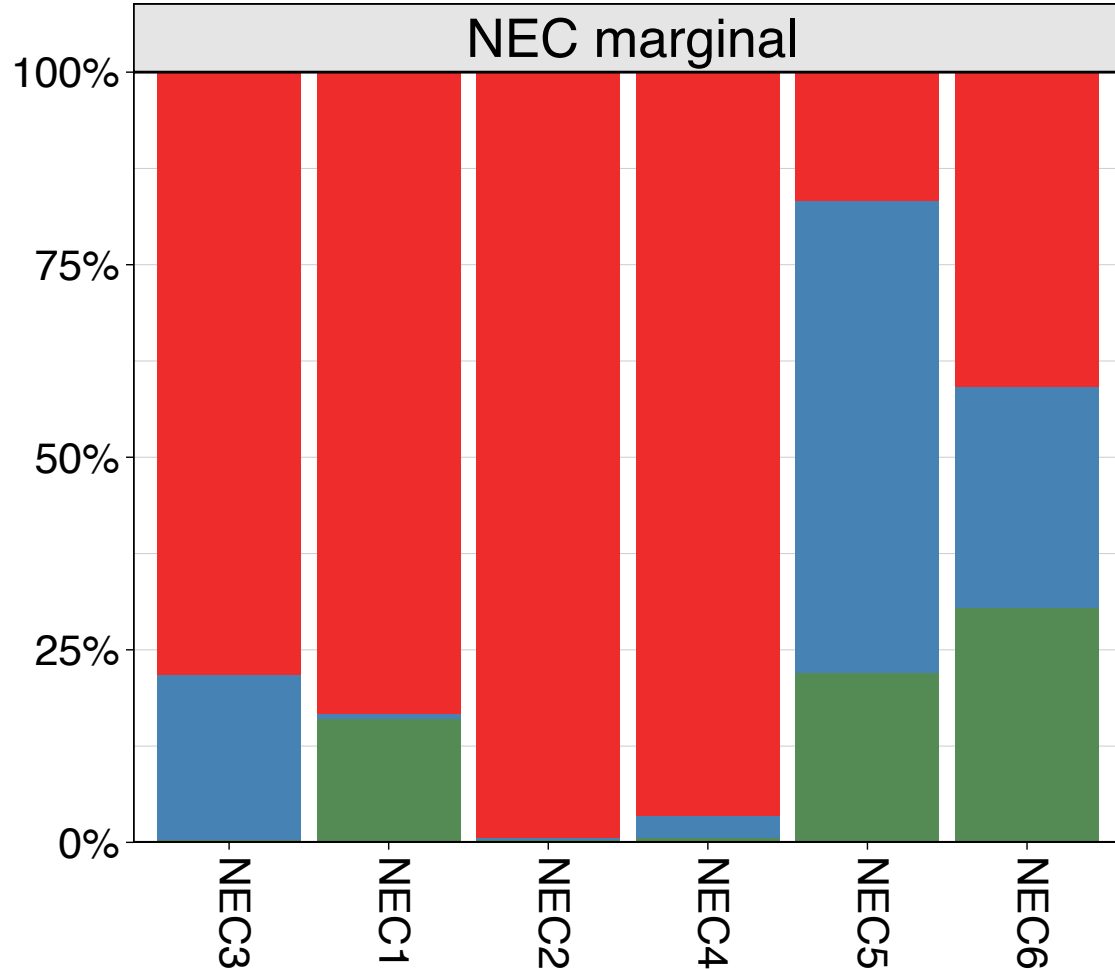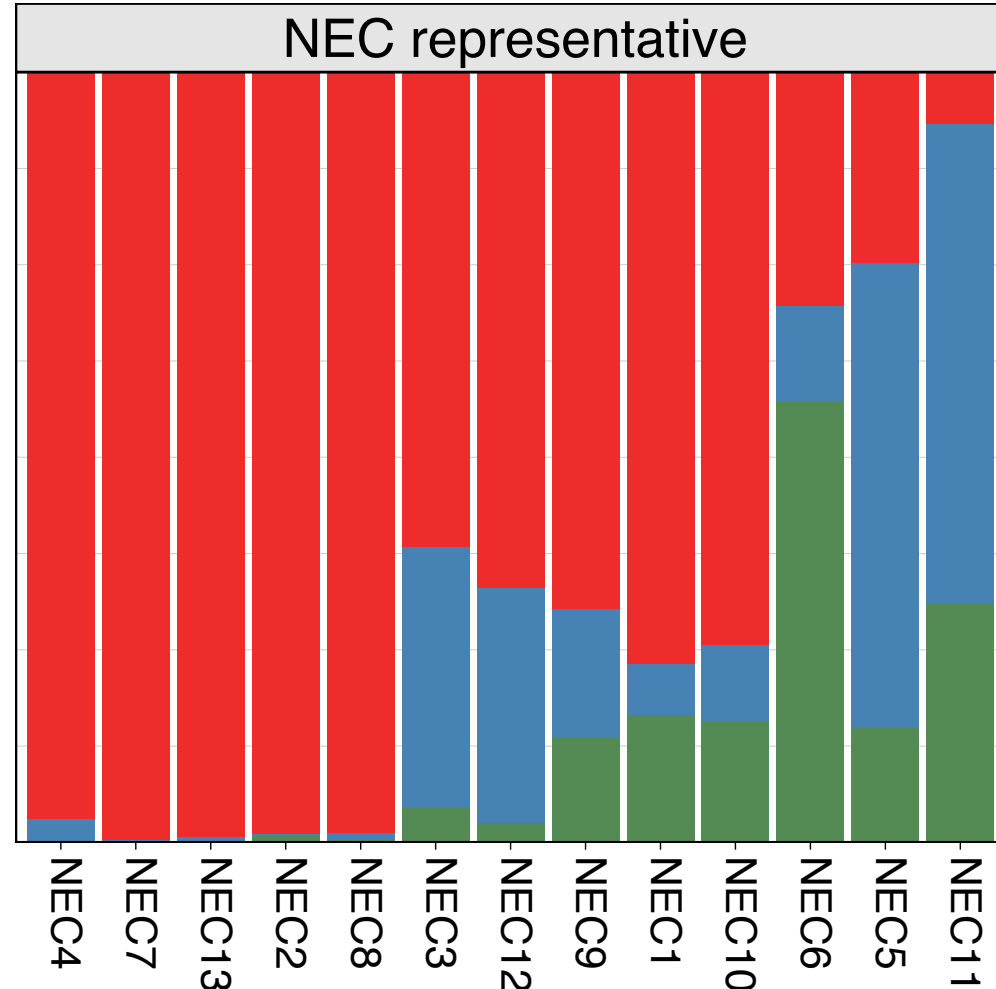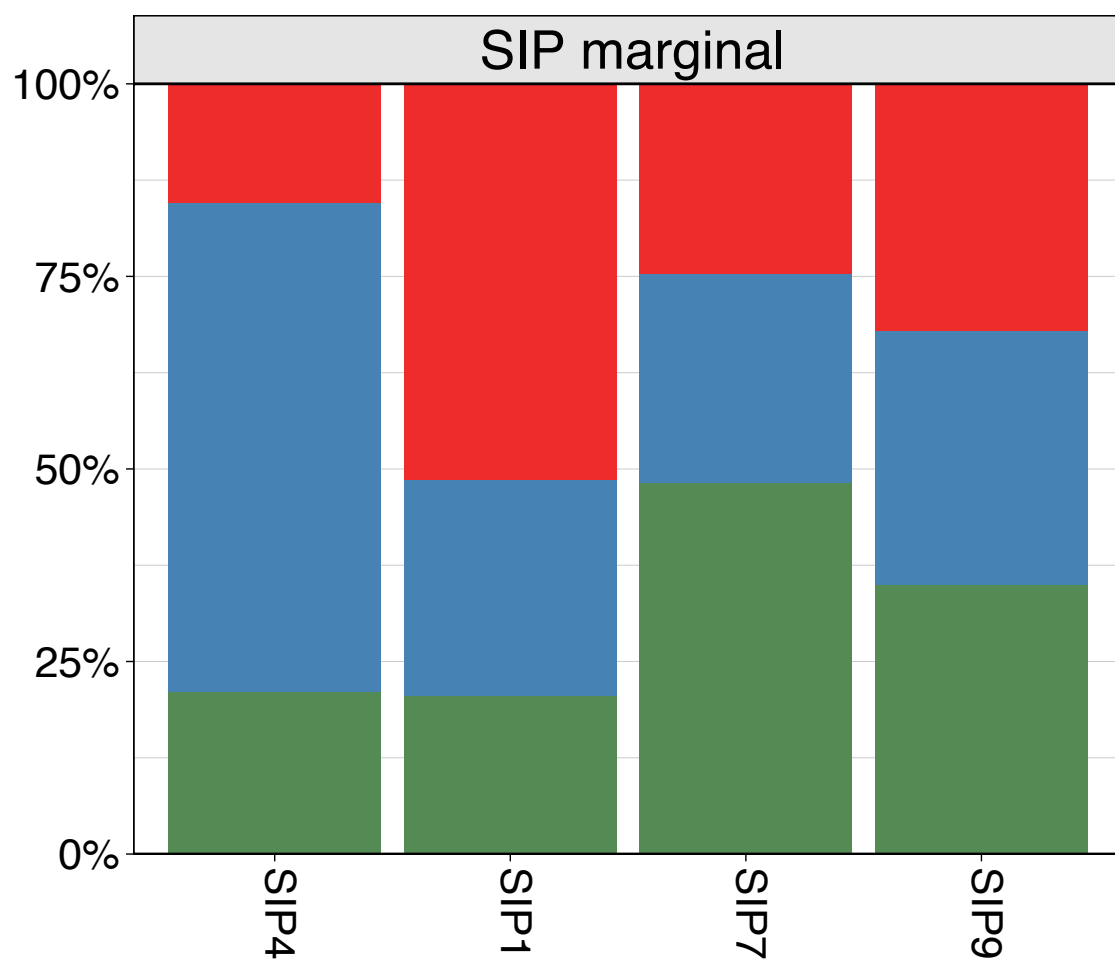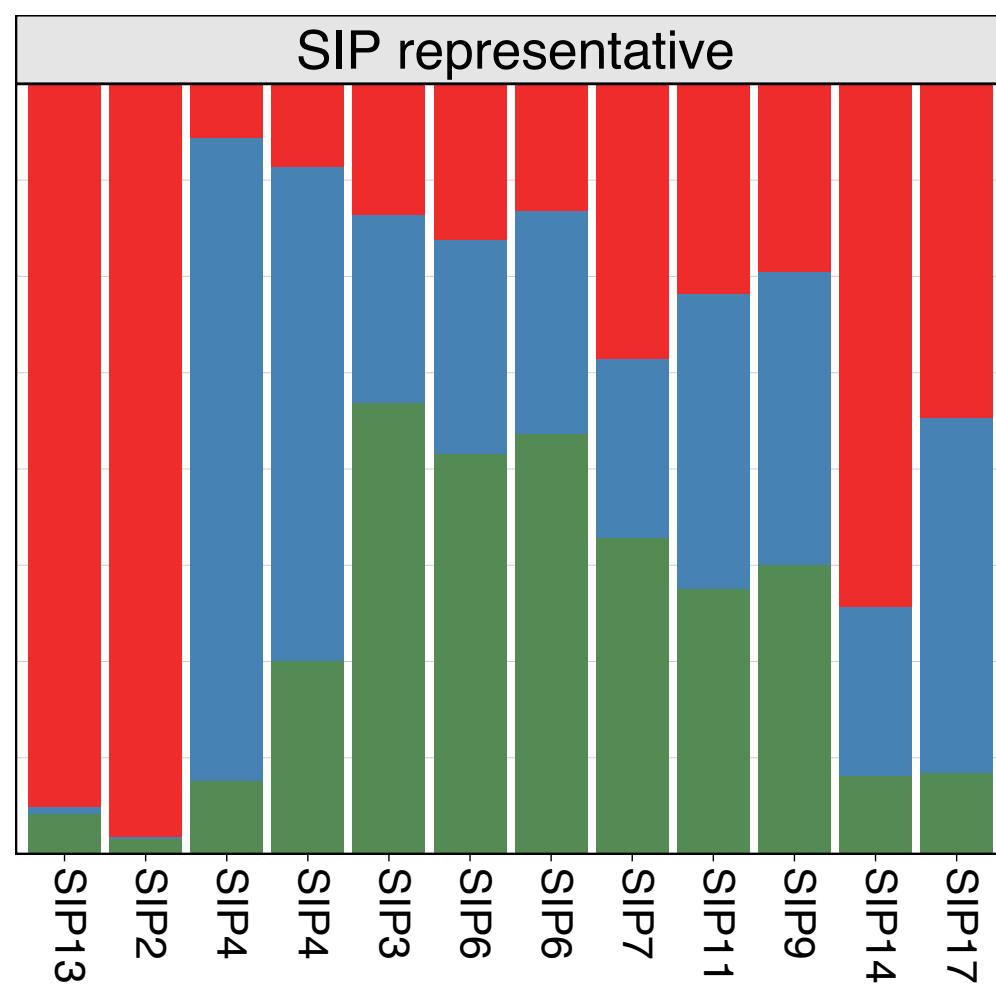

**Phylum**

- Proteobacteria
- Bacteroidetes
- Other

Supplement: Supplementary file 5 — Figure S3. Stacked bar plot of dominant phyla. Marginal and representative sections from each infant were included separated by necrotising enterocolitis (NEC) and spontaneous intestinal perforation (SIP). (PDF 169 kb) [file 12866_2019_1426_MOESM5_ESM.pdf]
